# Integrated network analysis reveals a novel role for the cell cycle in 2009 pandemic influenza virus-induced inflammation in macaque lungs

**DOI:** 10.1186/1752-0509-6-117

**Published:** 2012-08-31

**Authors:** Jason E Shoemaker, Satoshi Fukuyama, Amie J Eisfeld, Yukiko Muramoto, Shinji Watanabe, Tokiko Watanabe, Yukiko Matsuoka, Hiroaki Kitano, Yoshihiro Kawaoka

**Affiliations:** 1 ERATO Infection-Induced Host Responses Project, Saitama, 332-0012, Japan; 2 School of Veterinary Medicine, Department of Pathobiological Sciences, Influenza Research Institute, University of Wisconsin-Madison, Madison, WI, USA; 3 Division of Virology, Department of Microbiology and Immunology, Institute of Medical Science, University of Tokyo, Tokyo, Japan; 4 The Systems Biology Institute, Tokyo, Japan; 5 Division of Systems Biology, Cancer Institute, Tokyo, Japan; 6 Sony Computer Science Laboratories, Inc, Tokyo, Japan; 7 Okinawa Institute of Science and Technology, Okinawa, Japan; 8 Department of Special Pathogens, International Research Center for Infectious Diseases, Institute of Medical Science, University of Tokyo, Tokyo, 108-8639, Japan; 9 International Research Center for Infectious Diseases, Institute of Medical Science, University of Tokyo, Tokyo, 108-8639, Japan

**Keywords:** Influenza, Host response, Microarray, pH1N1, Systems biology

## Abstract

**Background:**

Annually, influenza A viruses circulate the world causing wide-spread sickness, economic loss, and death. One way to better defend against influenza virus-induced disease may be to develop novel host-based therapies, targeted at mitigating viral pathogenesis through the management of virus-dysregulated host functions. However, mechanisms that govern aberrant host responses to influenza virus infection remain incompletely understood. We previously showed that the pandemic H1N1 virus influenza A/California/04/2009 (H1N1; CA04) has enhanced pathogenicity in the lungs of cynomolgus macaques relative to a seasonal influenza virus isolate (A/Kawasaki/UTK-4/2009 (H1N1; KUTK4)).

**Results:**

Here, we used microarrays to identify host gene sequences that were highly differentially expressed (DE) in CA04-infected macaque lungs, and we employed a novel strategy – combining functional and pathway enrichment analyses, transcription factor binding site enrichment analysis and protein-protein interaction data – to create a CA04 differentially regulated host response network. This network describes enhanced viral RNA sensing, immune cell signaling and cell cycle arrest in CA04-infected lungs, and highlights a novel, putative role for the MYC-associated zinc finger (MAZ) transcription factor in regulating these processes.

**Conclusions:**

Our findings suggest that the enhanced pathology is the result of a prolonged immune response, despite successful virus clearance. Most interesting, we identify a mechanism which normally suppresses immune cell signaling and inflammation is ineffective in the pH1N1 virus infection; a dyregulatory event also associated with arthritis. This dysregulation offers several opportunities for developing strain-independent, immunomodulatory therapies to protect against future pandemics.

## Background

In April of 2009, a new pandemic H1N1 (pH1N1) influenza virus strain emerged in Mexico [1], and the subsequent global pandemic claimed more than 18,000 lives [2]. Human infections with pH1N1 tended to be mild with typical clinical symptoms (e.g., fever, sore throat, vomiting, and occasional diarrhea) [1]. However, in instances where death occurred, autopsies showed necrotizing bronchitis, a symptom that has also been observed in previous pandemics [3,4]. Loss of life during the 2009 pandemic was minor compared to other pandemics (e.g., the 1918 Spanish influenza pandemic, which caused an estimated 50 million deaths worldwide [5]); however, 2009 pH1N1 infections have resulted in more severe illness in young, previously healthy individuals, which is atypical for seasonal influenza virus isolates [6-8].

Currently licensed antiviral compounds against influenza virus (e.g., oseltamavir) interfere with specific functions of viral proteins and are one defense against seasonal and newly emerging pandemic influenza viruses. However, the sudden increase in oseltamivir-resistant seasonal H1N1 virus strains in 2007–2008 [9] and the existence of resistant pH1N1 virus isolates [10] strongly underscore the urgent need for the development of novel strategies to alleviate influenza virus-induced disease. As an alternative to directly inhibiting viral proteins, aspects of the host response that strongly correlate with host pathology could be targeted to reduce the overall severity of infection. Such therapies may be more robust against the emergence of drug-resistant strains because they do not target influenza virus-specific gene products, which are subject to drug-mediated selective pressure. Therefore, a better understanding of the mechanisms contributing to influenza virus pathogenesis is needed to provide the basis for the development of novel host-modulatory therapies. We have reported that a pH1N1 isolate (influenza A/California/04/2009 [H1N1], referred to as CA04) is more pathogenic than a seasonal H1N1 isolate in several mammalian models, including cynomolgus macaques [11]. Specifically, CA04-infected macaques exhibited higher body temperatures, more efficient virus replication in both the upper respiratory tract and lungs, increased production of pro-inflammatory cytokines (e.g., CCL2 and IL6), and more severe lung lesions at both early (3 days) and late (7 days) times post-infection. In addition, we observed an increased presence of inflammatory infiltrates in alveolar spaces and more abundant viral antigen staining of both type I and type II pneumocytes. These pathological characteristics are similar to those that distinguish highly pathogenic avian H5N1 and 1918 influenza viruses from lower pathogenicity isolates [12-14], suggesting a common host response theme that may contribute to increased pathogenicity phenotypes for diverse influenza viruses. However, the specific mechanisms underlying the severity of CA04 virus pathogenicity remain to be fully elucidated.

In this study, we undertook a unique bioinformatics approach to identify host processes that might be responsible for the enhanced pathology of CA04 in macaques. Other studies have used microarray analysis in conjunction with bioinformatics tools (e.g., Ingenuity Pathway Analysis (IPA) or DAVID [15]) to dissect the response to influenza virus infection, primarily at the level of the outcome of host signaling (i.e., differentially expressed transcripts) [13,14,16-18]. However, these studies typically have used such tools in isolation, and largely have not attempted to predict causative regulatory influences mediated by factors existing outside of the primary dataset. Here, we have combined process-level and cell-specific functional enrichment analyses of highly differentially expressed transcripts in CA04-infected lung tissues, identification of putative transcription factors involved in regulating differential expression, canonical pathway enrichment, and protein-protein interactions to develop an integrated pathogenicity-associated host response network. This novel approach revealed not only differentially regulated pathways and differences in immune cell populations that correlate with enhanced CA04 pathogenicity, but importantly, also identified critical regulatory intermediaries between these correlates. We suggest that the resulting network can be systematically explored for novel therapeutic intervention.

## Results

### CA04 may promote inflammation through increased expression of chemotactic molecules and caspase-1 induction and suppression of glutathione S-transferase (GST) expression

The CA04 virus induces more severe lesions in the lungs of cynomolgus macaques relative to a seasonal influenza virus isolate (A/Kawasaki/UTK-4/09 [H1N1; referred to KUTK4]) [11]. To identify host functions and pathway activity that might explain this difference, we compared the global transcriptional response of CA04- and KUTK4-infected lung tissues derived from the animals reported in [11]. Microarray analysis of RNA isolated from within gross lesions identified 101 differentially expressed (DE; See Methods) transcripts between the two infections on day 3 p.i. and 854 DE transcripts on day 7 p.i. More than half of the DE transcripts from day 3 were also DE on day 7, and the overlap between time points was significant (Fisher’s Exact test, *P* < 10^−16^). A table summarizing the DE test results is shown in Additional file 1, and total lists of DE transcripts from days 3 and 7 are available in Additional file 2. It is important to note that, while lung lesion severity varied between samples (Additional file 3), principle component analysis did not identify any relationships between the samples based on lesion severity (data not shown).

**Figure 1:**
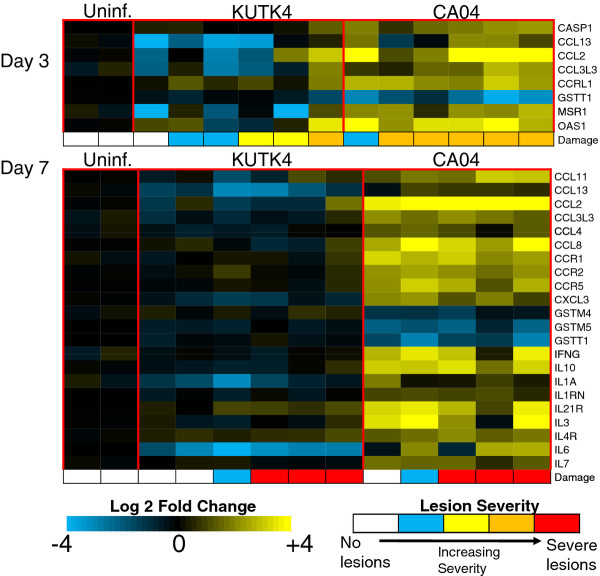
**CA04 differentially expressed genes.** The transcription profiles of select DE genes on day 3 (top) and day 7 p.i (bottom). Each column represents the gene expression profile of an individual macaque lung tissue sample; gene expression levels for two samples acquired from a uninfected animal are also shown (to the left) for reference. The lesion severity, as described in (11), for each sample is shown at the bottom (Damage) of each heat map. White indicates no lesions; blue represents lung lesions with severe alveolar wall thickening; yellow indicates mild lung lesions with some antigen-positive cells present; orange indicates severe lesions containing a significant number of virus antigen-positive cells; and red indicates severe lung lesions in which the alveolar space was filled with inflammatory cells.

We first focused on genes commonly associated with inflammation and apoptosis (Figure 1). Enhanced up-regulation of interferon (IFN)-stimulated genes (ISGs) was observed on day 3 and day 7 p.i., indicating that CA04 induced more robust type I IFN signaling than did KUTK4 (Additional file 1). On day 3 p.i., three chemokines (CCL13, CCL2 and CCL3L3) were more up-regulated with CA04 infection; and these together with four others (CCL11, CCL4, CCL8 and CXCL3) were also more up-regulated on day 7. In general, the up-regulated chemokines perform pleiotropic recruitment activities against multiple immune cell subtypes; however, CCL2 and CCL3L3 exhibit potent chemotactic activity toward monocytes [19,20]. On day 7, we also observed enhanced expression of chemokine receptors that are abundant on monocytes (CCR1 and CCR2), interleukins that are primarily expressed and secreted by macrophages (IL10 and IL1A) and molecules that regulate lymphocyte activities (IL21R, IL4R, and IL7). Collectively, these observations are consistent with detailed histopathology analysis of immune cell infiltrates in macaque lungs infected with a similar pH1N1 strain [7], and support the suggested involvement of monocyte and lymphocyte sub-populations in the regulation of CA04 mediated pathology.

Particularly interesting was the observed up-regulation of caspase 1 (CASP1) in CA04-infected lungs. CASP1 is converted to its active form by the inflammasome signaling complex, which is activated by influenza virus infection [21,22]. In turn, CASP1 cleaves latent IL1β and IL18 leading to the secretion of the activated forms of these pro-inflammatory cytokines [23]. Consistent with increased CASP1 expression, IL1β and IL18 protein concentrations were elevated in the lungs of 2 of 3 CA04-infected macaques at day 7 (see Additional files in [11]). Taken together, these observations are indicative of increased inflammasome activity in CA04 infections.

Among the CA04-down-regulated transcripts, we observed several members of the cytosolic glutathione S-transferase (GST) protein family (GSTT1 on day 3; GSTT1, GSTM5, and GSTM4 on day 7). GSTs are antioxidant enzymes that participate in the inactivation of secondary metabolites formed as a result of oxidative stress, and thereby serve a protective role in the cell [24]. Similar suppression of GST transcription was observed in the kidneys of chickens infected with an HPAI virus strain [25], and it has been suggested that oxidative stress may be a key pathway involved in lung injury associated with HPAI virus infection [26]. Thus, the CA04-enhanced down-regulation of GST likely promotes oxidative injury in lung tissue.

### CA04-infected macaques exhibit enhanced inflammatory and cell cycle gene expression

**Figure 2:**
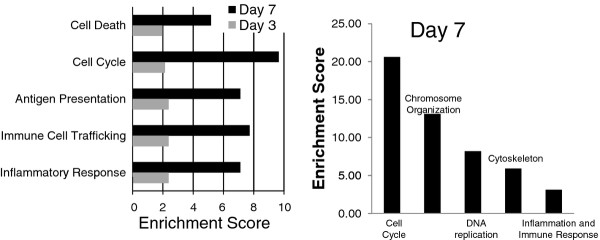
**The enriched biological functions of up-regulated genes, as determined by (left) IPA and (right) DAVID.** The IPA enrichment score is reported as the -log10 of the FDR-adjusted P-value. DAVID clusters redundant or highly related annotations into clusters and reports the enrichment as the –log10 of the average P-value of the terms in each annotation cluster. DAVID did not identify any significant annotations for genes which were up-regulated on day 3, and only enrichments for day 7 are shown.

IPA and DAVID were next used to identify functional differences between CA04 and KUTK4 infections (Figure 2). IPA uses Fisher’s Exact test to determine the enrichment significance for each function annotated in the IPA database. DAVID, on the other hand, uses clusters of related annotations built from several annotation databases (e.g., gene ontology, pathway data), and calculates the enrichment score of a cluster by averaging unadjusted *P*-values (determined by Fisher’s Exact test) of the annotations within the cluster [15]. We reasoned that analyzing the CA04 pathogenicity-associated DE transcripts using both methods would provide increased functional information and/or cross-validation between methods. DE transcripts were separated into up-regulated or down-regulated groups (CA04 expression relative to KUTK4 expression) for each time point and analyzed separately. All significantly enriched (FDR-adjusted *P*-value < 0.01) IPA functional annotations and DAVID functional clusters are included in Additional files 4 and 5, respectively.

IPA analysis of up-regulated DE transcript identified enrichment for ‘Inflammatory Response’, ‘Immune Cell Trafficking’, and ‘Antigen Presentation’ at day 3 p.i., consistent with the increased immune cell infiltrates observed in the original pathology examination [11]; no significant clusters were identified in this time point using DAVID analysis (Figure 2). Transcripts up-regulated on day 7 were enriched for many of the same IPA categories found on day 3, and both IPA and DAVID identified ‘Cell Cycle’ as being the most highly enriched, up-regulated process on day 7. More specific categories, such as positive or negative regulation of cell cycle, did not provide conclusive evidence as to the effect of the regulation on cell cycle (see Additional files 5 and 6). Among the downregulated transcripts, we saw enrichment only with IPA at the 7 d p.i. time point, and only in two categories with no obvious connection to the host response to infection (e.g., ‘Psychological Disorders’ and ‘Neurological Diseases’) (Additional file 4). These results suggest that functional differences between CA04 and KUTK4 initiate early after infection and are amplified later in the infection, while both analyses identified the cell cycle as the most prominently enriched functional annotation for transcripts that were highly DE with CA04 infection.

### CA04 infection enhances gene expression associated with both early/innate and late/adaptive immune cell responses

**Figure 3:**
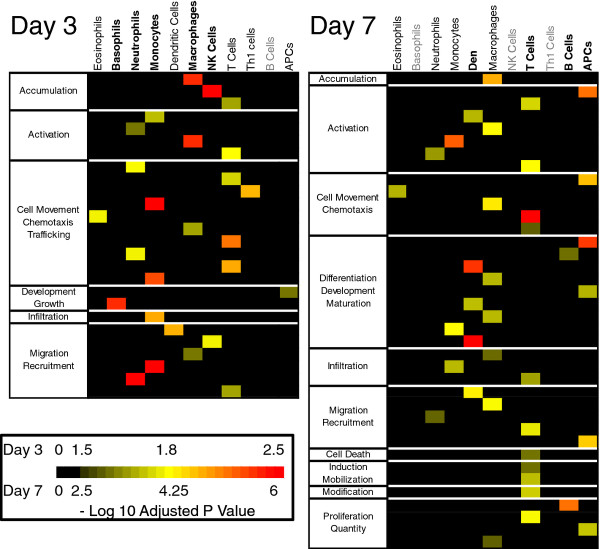
**Cell-specific CA04-induced functional enrichment.** Cell-type specific enrichment of CA04-induced up-regulated DE transcripts is shown for day 3 (left) and day 7 (right) post-infection. Enriched cell-type-specific IPA annotations (FDR-adjusted *P*-value < 0.05) were sorted according to function (categories shown to the left of each panel) and cell-type (shown at the top of each panel), and the level of enrichment was illustrated by a heat map showing the -log_10_-adjusted *P*-value for each annotation. Because *P*-values associated with highly related annotations (e.g., ‘Cell Movement’, ‘Chemotaxis’ and ‘Trafficking’) were grouped together, a cell type could be enriched several times within a particular category. Cell types with the highest enriched categories are shown in boldface (top), whereas cell types for which no categories were enriched are shown in grey. All other cell types, exhibiting intermediate levels of enrichment in various categories, are represented in normal black text. A color-key for the heat map is shown at the lower left of the figure, in which day 3 *P*-values are represented by the range above the color bar and day 7 *P*-values are indicated below the bar.

As differences in immune cell subtypes can also be key regulators of pathology, we performed a cell-specific, functional analysis of DE genes by using IPA’s specialized functional annotations for disease and discovery. In addition to providing a general description of function for a category of enriched genes, this approach captures details such as the cell or tissue type in which the gene exerts its function and the orientation of regulation. For down-regulated genes on both days, few categories satisfied our enrichment criteria (FDR-adjusted *P* < 0.05), and no immune cell-specific annotations were enriched. In contrast, the up-regulated gene lists produced more than 500 enriched categories for both days, many of which exhibited functions pertaining to the activation, chemoattraction, infiltration and development of specific immune cell subtypes (a complete list of significant categories for up- and down-regulated genes is shown in Additional file 6). To obtain a clearer representation of how CA04-induced gene expression influences different immune cell sub-types, we categorized enrichment by cell-type and function and employed a heat map to show how the enrichment was distributed in relation to time (summarized in Figure 3; see also Additional files 7 and 8 for a detailed heat map). On day 3, the immune cell subtype most prominently affected by CA04-enhanced gene expression was macrophages, for which categories related to activation, accumulation, and chemotaxis were very highly enriched (Figure 3). T lymphocytes and neutrophils were also broadly affected at this time point, although annotations were not as significant as they were for macrophages. In addition, some enrichment was observed for genes affecting the migration of dendritic cells, eosinophils, and natural killer cells. On day 7, no significant terms related to natural killer cells or basophils were observed, and enrichment of the activation and migration of neutrophils was quite minor, demonstrating that several aspects of the innate immune response had tapered. Instead, we found high enrichment for B lymphocyte proliferation and broad enrichment in categories associated with T lymphocyte chemotaxis, infiltration, proliferation, activation, and death. In addition, several functions of antigen presenting cells (APCs) were more up-regulated in the CA04 infection. The original pathology results [11] did not specify the cell types that comprised the enhanced inflammatory infiltrates on day 3 in CA04-infected lung tissue, but they did identify an increase in APCs on day 7 p.i. [11]. These data point to an increased presence and activation of primarily innate immune cell subtypes early after infection, followed by enhanced influx and activity of APCs and adaptive immune cell types in CA04-infected macaque lungs.

### Inflammatory and apoptotic transcription factor binding sites are enriched in CA04-specific DE genes

Next, transcription factor activity was determined by identifying transcription factors whose promoter sequences were highly enriched among the genes that were DE between the CA04 and KUTK4 infections. We used the GATHER software tool [27] which matches transcription factors with their experimentally proven binding sites and determines whether there is significant enrichment of a transcription factor binding site within a given gene list. The transcription factors that matched the most highly enriched promoter sequences for each time point after infection are shown in Table 1.

**Table 1:** Top three transcription factors enriched for genes differentially expressed (DE) on day 3 or day 7 post-infection between CA04 virus-infected and KUTK4 virus-infected lung tissue

	**Transcription factor**	% DE genes*	**-ln (*P*Value)**
Day 3	Interferon Regulatory Factor 7 (IRF7)	55.8	3.54
	Forkhead box P3 (FOXP3)	53.8	4.08
	Hepatic nuclear factor 1 (HNF1A)	42.3	7.18
Day 7	MYC-associated zinc finger protein (MAZ)	74.6	6.05
	Nuclear transcription factor Y alpha (NFYA)	24.4	8.31
	Paired box 5 (PAX5)	15.7	5.56

* The percentage of genes DE on each day that were regulated by each transcription, according to GATHER®.

Day 3 promoter sequence enrichment identified interferon regulatory factor 7 (IRF7), forkhead box P3 (FOXP3), and hepatic nuclear factor 1 (HNF1A). IRF7 is activated by toll-like receptors and RIG-I, and its increased activity likely reflects the increased replication of CA04 in macaque lungs observed on day 3 p.i. [11]. FOXP3 is a transcription factor specific to a subset of regulatory T lymphocytes that are known for suppressing inflammatory reactions [28]. FOXP3 promoter site enrichment thus implicates regulatory T lymphocytes in the early phase of CA04 infection, possibly reflecting an increased requirement to confront the CA04-enhanced inflammatory response. HNF1A has a wide range of functions, but recent evidence suggests that it may serve as a link between metabolic and inflammatory pathways involved in atherosclerosis [29].

On day 7, nuclear transcription factor Y alpha (NFYA), paired box 5 (PAX5) and MYC-associated zinc finger protein (MAZ) transcription factor activities were highly significant. NFYA directly induces transcription of many pro-apoptotic genes and is implicated in p53-mediated apoptosis [30,31]. To determine if there was any significant association between the genes regulated by NFYA and the genes annotated with ‘Cell Death’ in IPA, we used a one-sided Fisher’s exact test, but we did not observe significant overlap between these gene sets (only 13.9%, *P* = 0.18, of the DE genes that were annotated for ‘Cell Death’ were also regulated by NFYA; further limiting the gene set to only upregulated genes resulted in a non-significant overlap of 22.0%, *P* = 0.69). PAX5 is a specific marker for B cells [32], and its enrichment is consistent with the enhanced B cell presence and activity suggested by our cell type-specific functional enrichment analysis for day 7 (Figure 3). The enriched transcription factor affecting the greatest percentage of CA04 DE genes was MAZ, whose regulation is strongly correlated to inflammation and other common inflammatory markers (e.g., IL6, IL1β) [33]. Significant overlap was identified between the genes annotated for ‘Inflammation Response’ by IPA and genes that contained the MAZ binding sequence, when considering all DE genes on day 7 (72.3% overlap, *P* = 0.05), but the overlap was not significant when only considering genes upregulated on day 7 (63.9% overlap, *P* = 0.25). But within the set of upregulated genes, there was a very significant overlap between the MAZ regulated gene set and the set of genes annotated with “Cell cycle” in IPA (74.5% overlap, *P* = 0.003). This suggests that MAZ is closely associated to cell cycle activity despite its established role in inflammation. Overall, transcription factor enrichment analysis identified several factors involved in the regulation of inflammatory processes, apoptosis and cell cycle, and is consistent with the enriched functional processes observed among the genes that were more DE with CA04 infection.

### Network analysis identifies the primary gene expression moderators in CA04-infected tissue

Thus far, we have used enrichment analyses to isolate individual processes differently regulated by the CA04 virus. Next, we applied a network approach to determine the regulatory interactions that may be coordinating gene expression. Within the IPA-curated human protein-protein interaction (PPI) network, we searched for subnetworks that were highly populated with DE genes, and then we used the protein degree (i.e., the “centrality”, or the number of interactions a protein has) to identify highly connected hub proteins within each subnetwork. Hub proteins are often more essential than non-hub proteins [34,35] and may play critical roles in regulating the network’s overall biological function [36].

**Figure 4:**
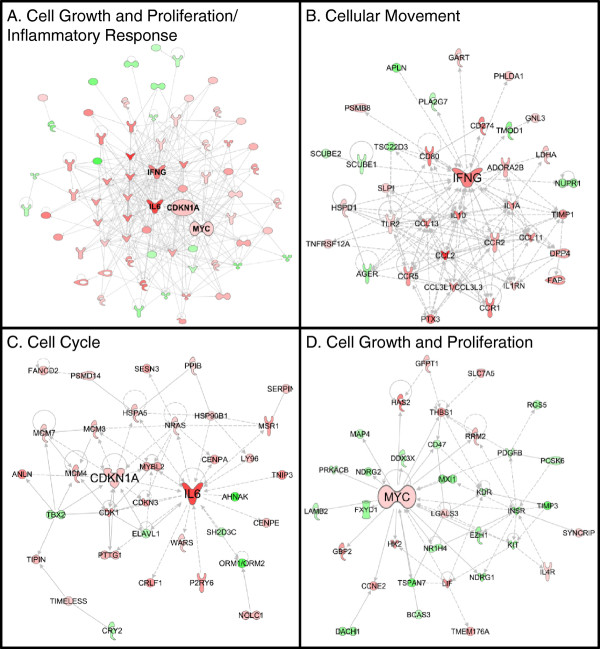
**Protein-protein interaction subnetworks differentially regulated in CA04-infected lung tissue.** IPA was used to determine subnetworks within the human PPI which were highly populated with genes differentially expressed in CA04-infected tissue on day 7 p.i. We show the top networks (based on their network score) when **(A)** up to 70 proteins are allowed in the subnetwork and **(B-D)** the top 3 networks when the size of the subnetwork is limited to 35 proteins. Each network is labeled according to its most enriched biological function. Proteins that’s transcript is up-regulated or down-regulated in CA04-infected lung relative to KUTK4-infected lung are colored red and green, respectively. Additional file 9 provides a larger illustration of panel A with all protein labels shown.

An initial assessment allowing for a network size of up to 70 members did not identify any significant subnetworks at 3 d.p.i.; however, several were detected among DE genes on day 7. The most significant of these was highly enriched for ‘Cell Growth and Proliferation’ and ‘Inflammatory Response’ annotations (Fisher’s exact test *P*-values < 10^-15^) and was centered on four hub genes: IFNG, MYC, IL6, CDKN1A (Figure 4A, a more detailed illustration in Additional file 9). A more restricted analysis – limiting the number of subnetwork members to 35 genes – recapitulated this result, yielding three smaller subnetworks with the same hub genes, each enriched for unique biological functions (Figure 4B-D). IFNG, an antiviral cytokine with potent macrophage activation activity and immunoregulatory functions in adaptive immunity, exhibited high centrality in a largely upregulated subnetwork that was enriched for ‘Cellular Movement’ (*P-*value = 3.96E-16) (Figure 4B). Notably, 8 of the 35 proteins in this subnetwork were also members of the ‘Role of Cytokines in Mediating Communication between Immune Cells’ canonical pathway (Additional file 10), thus implicating IFNG as a putative regulator of the increased late adaptive immune response in CA04 infections. The second subnetwork, consisting primarily of upregulated genes, was highly enriched for ‘Cell Cycle’ functions (*P-*values = 5.4E-7) (Figure 4C). Interestingly, both the pro-inflammatory cytokine, IL6, and the cyclin-dependent kinase inhibitor, CDKN1A, appeared as hubs within this subnetwork, suggesting a novel connection between the host inflammatory response and the highly enriched, cell cycle-associated processes that occur in the late stage of infection. CDKNIA’s involvement further suggests that the broad category of “cell cycle” is likely to be more specifically related to cell cycle arrest, since CDKN1A is a known inhibitor of cell cycle.

The final network (Figure 4D) is centered around the MYC transcription factor, which was up-regulated in CA04 infections, and enriched for ‘Cell Growth and Proliferation’ (*P-*value = 2.0E-6). Generally, MYC activity is associated with cell proliferation, but MYC overexpression has also been associated with cell cycle arrest [37]. As the majority of the genes in this subnetwork were down-regulated in the CA04 infection, this suggests that cell proliferation is being suppressed. Therefore, network analysis results were consistent with previous enrichment analyses, and further suggested that IFNG, IL6 and CDKN1A may play prominent roles in regulating the severity of CA04-associated disease relative to seasonal influenza virus.

### The CA04-KUTK4 differentially active immune network

Finally, we integrated the cell type enrichment and promoter sequence enrichment analysis with the results of the subnetwork analysis into a coherent map of the CA04-induced immune response. To better clarify how critically positioned hub proteins affected canonical pathways, we performed pathway enrichment and selected two significantly enriched pathways – the ‘Role of Cytokines in Mediating Communication between Immune Cells’ (Additional file 10) and the ‘Role of CHK Proteins in Cell Cycle Checkpoint Control’ (Additional file 11) – for further examination. These pathways were chosen because (i) they contained several of the genes that mapped to the subnetworks shown in Figure 4, (ii) the function of these pathways matched the function of the subnetworks and (iii) the pathway was highly enriched for genes DE in CA04-infected lung (FDR-adjusted *P* < 0.05, Additional file 12 provided details on the pathway enrichment analysis). Furthermore, these pathways contain cell-specific expression information for many of the genes involved in this study.

**Figure 5:**
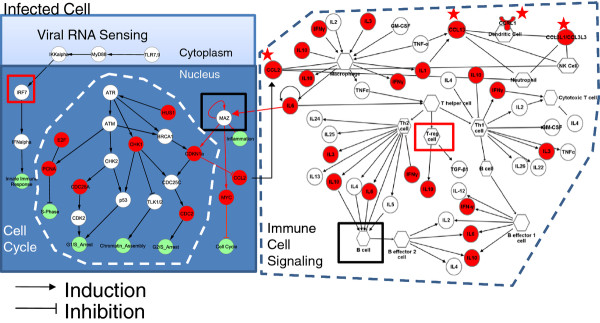
**Differential response network between CA04- and KUTK4-infected lung tissue.** Highly enriched canonical pathways identified by using IPA (namely, ‘Role of CHK Proteins in Cell Cycle Checkpoint Control’, enclosed by the white, broken line and ‘Role of Cytokines in Mediating Communication between Immune Cells’, enclosed by the blue broken line) were integrated by using highly enriched transcription factors and IPA's Expert Curated protein-protein interaction database. Newly identified interactions which are not part of the canonical pathways are illustrated with red arrows, whereas interactions belonging to the canonical pathways are colored black. Proteins that are differentially up-regulated in CA04-infected lung tissue relative to KUTK4-infected lung tissue on day 7 p.i. are colored red. Three chemokines and one chemokine receptor which were up-regulated in the CA04 infection on day 3 as well are demarcated by red stars adjacent the protein. Transcription factors or cell types for which the transcription factors surrogate as cell markers are boxed in red or black representing enrichment on day 3 or day 7 p.i., respectively.

Interactions within each pathway were diagrammed, taking into consideration activating and inhibitory relationships (Figure 5). Next, we searched the IPA PPI database for interactions linking the pathways, and we identified the MAZ transcription factor, which, incidentally, was also one of the most enriched transcription factors in the promoter enrichment analysis. Specifically, MAZ activates expression of CDKN1A (a component of the CHK pathway and a ‘Cell Cycle’ subnetwork hub), thereby controlling cell cycle progression through the G1 checkpoint [38] (see also Figure 5). MAZ is also activated by both IL1 and IL6 through MAP kinase-dependent phosphorylation in human cells [39] and is a member of the MYC complex. Thus, MAZ, in a network context, appears to be a critical intermediary between the identified subnetworks and potentially their biological function.

The newly identified interactions were added to the previously mentioned pathway diagrams, and gene expression and differentially active transcription factors were overlaid to depict the CA04-induced immune response (Figure 5). The viral RNA sensing pathway was added since IRF7 was found to be active in the CA04-infection on day 3 [40]. The precise cell-type in which cell cycle arrest and MAZ activity occurs is an open question; therefore, the specific type of infected cell was not clarified in the network, and was labeled only as ‘Infected Cell’. CA04-enhanced DE genes were colored red, and differentially active transcription factors or the cell types their differential activity may represent (e.g., FOX3P is regulatory T lymphocyte-specific) were boxed in red (if enriched on day 3 p.i.) or black (if enriched on day 7 p.i.). The full names and Entrez IDs for all genes depicted in Figure 5 are listed in Additional file 13.

In this network, only four genes (all chemokines) were up-regulated on day 3 p.i. (demarcated by red stars) and their primary role is the chemoattraction of innate immune cell types (NK cells, neutrophils, macrophages, and dendritic cells). The activity of IRF7 and regulatory T lymphocytes (implicated by FOX3P enrichment) was enhanced on day 3, as evidenced by the promoter sequence enrichment (Table 1). Major differences in the immune response network appeared on day 7 p.i., with upregulation of additional chemokines and cytokines responsible for the activation and differentiation of innate and adaptive immune cells (see the non-starred red nodes on the right panel of Figure 5). In particular, interferon gamma (IFNG), which is important for cytotoxic T cell function and elimination of virus-infected cells, was up-regulated (Figure 5, right). Additionally, upregulation of IL6 and IL10 (Figure 1) can lead to B lymphocyte activation, while the presence of B lymphocytes was supported by the enrichment of the PAX5 promoter sequence (Table 1). Increased immune cell presence and continued IL6 (and/or IL1) production would allow the simultaneous upregulation of MAZ activity, thereby impacting inflammatory and cell cycle signaling through CDKN1A transcription (Figure 5, see the MAZ transcription factor node and its incoming and outgoing edges). The cell cycle is further impacted by MAZ’s interaction with MYC, which we suspect is involved in cell cycle arrest, since many of MYC’s target proteins were downregulated during infection (Figure 4D). Overall, our data identify a novel, transcriptionally active link between influenza-induced inflammation and cell cycle arrest (i.e., the MAZ transcription factor) and suggest that inflammation-induced, MAZ-dependent cell cycle disruption may be responsible, at least in part, for apoptosis and tissue injury related to influenza virus pathogenicity.

## Discussion

GO and pathway enrichment studies can detail many key aspects of the host response, but these biological functions and processes must be integrated to create host response models capable of linking the effects of transcription to the molecular and signaling events driving those effects. The differentially regulated host response network presented here represents a novel effort to combine various, independent analyses of DE genes into a coherent protein-protein/protein-cell type interaction architecture. This approach allowed us to link immune cells and their inflammatory activities with cell cycle regulation through the identification of a transcription factor (i.e., MAZ) that acts as an intermediary between these functional correlates of pH1N1 pathogenicity. While many studies have used microarrays to identify global transcriptional changes that characterize the host response to different influenza viruses in different systems, our integrated approach allows for a more specific understanding of the mechanisms of influenza virus-induced pathogenicity.

Prior to developing the differential host response network, we validated our transcriptional data by showing that it was, indeed, indicative of the pathological differences between the two infections (Figures 2 and 3). Initial functional enrichment results confirmed that the biological processes observed during the pathology examination (i.e., enhanced inflammation and increased immune cell infiltrates [11]) were also detectable in the transcriptional differences between CA04- and KUTK4-infected lung tissue. Therefore, it seems very likely that any additional functionality or pathway information derived from these data should have a highly correlative, if not causal, relationship with the enhanced pathology of the CA04 infection.

The enhanced ability of the CA04 virus to replicate in lung tissue does not lead to large differences in the host response on day 3 of the infection. The enhanced IRF7 transcription factor activity in CA04-infected lung is consistent with the increased toll-like receptor/RIG-I signaling one expects when there are increased levels of viral single-stranded RNA present in a sample. Enhanced chemoattraction of NK cells, dendritic cells, neutrophils, and macrophages is consistent with the enhanced inflammatory infiltrates observed on histopathologic examination. Most interesting is the implied difference in regulatory T lymphocyte populations, evident from FOXP3 promoter site enrichment, and the potentially enhanced IL-1β expression as a result of greater up-regulation of CASP1 in the CA04 infection. Regulatory T lymphocytes manage immune system homeostasis, and imbalances in T cell populations are often associated with increased inflammation and immune-mediated cell death [41,42]. The implicated regulatory T lymphocyte population change may be a factor in the enhanced inflammation and cell damage observed in CA04-infected lung tissue on day 3 p.i. Additionally, increased CASP1 induction of IL-1β could further promote inflammation in CA04-infected tissue.

In a previous study, lung samples infected with a highly pathogenic and mildly pathogenic pH1N1 virus were compared to KUTK4-infected tissue on day 1 p.i., and similar to the work presented here, enhanced inflammation and immune cell infiltration were identified as correlates of increased pH1N1 pathogenicity [7]. This study found NFκB mediated transcription as a potential mechanism of enhanced pathogenesis, but the degree to which the observed gene regulation was independent of viral replication is unclear. Furthermore, of the 101 transcripts DE on day 3 of our study, only 11 were also DE in the previous work. While this does represent a significant overlap (Fisher Exact test; P < 0.001), none of the genes identified as DE early in pH1N1-infected lung tissue in both studies are related to the immune response. In all, both studies show that early in the course of the infection, there is no obvious dysregulation of the host response with the potential exception of an imbalance in regulatory T lymphocytes, noted above.

By day 7 p.i., several mechanisms up-regulated in CA04-infected lung tissue can account for the continued enhanced pathology [11]. Lung tissue infected with CA04 on day 7 p.i. showed sustained activation and accumulation of immune cells despite the absence of replicating virus. In addition to the increased immune cell signaling and the activation of the adaptive immune response (evident by the B cell-specific PAX5 promoter site enrichment and GO enrichment analysis shown in Figure 3) we observed increased enrichment for cell cycle arrest. Cell cycle arrest has a complicated relationship with virus replication and the immune response. Arrest during G1 phase promotes greater influenza virus replication [43], but cell cycle arrest also often occurs in cells prior to apoptosis [44]. Given that cell cycle arrest was observed late in the infection, this is likely to be a host-controlled event, possibly reflecting severely damaged host cells selecting an apoptotic fate.

The MAZ transcription factor activity (Table 1) adds an additional layer of complexity between influenza infection-induced apoptosis, immune cell trafficking, and the observed cell cycle arrest. MAZ increases cyclin-dependent kinase inhibitor 1A (CDKN1A) expression [38] and is known to regulate MYC transcription [45] – two molecules with seemingly opposed effects on cell cycle. Increased CDKN1A transcription leads to cell cycle arrest and the production of serum amyloid A (SAA), which in turn leads to increased recruitment of immune cells to inflammatory sites [38,39,46]. Increased MYC transcription is most often associated with increased proliferation but it has also been linked to increased cell cycle arrest in fibroblasts [37]. Since the cell proliferation enriched subnetwork holds MYC in a highly central position (Figure 4D), the evidence suggests that the increased MAZ transcription is simultaneously inducing cell cycle arrest via CDKN1A and MYC pathways. Since lymphocytes typically proliferate in organized lymphoid tissue (e.g., in lymph nodes) and our samples were collected from within infected lung lesions, we suggest that regulation of cell cycle gene expression primarily occurs in infected epithelium or pneumocytes. Lastly, the fact that virus could not be isolated on day 7 for CA04 infected-lung tissue suggests that activation of the MAZ pathway may be in response to an overly aggressive immune response rather than virus replication. Further, while MAZ protein levels are directly correlated with chronic inflammation, the anti-inflammatory suppression of CCL2 transcription by CDKN1A [47] was not observed in our microarray data. Thus, there are multiple interactions involving MAZ which we feel are suitable targets to mitigate inflammation during moderate to highly pathogenic influenza infections. Further study validating the significance of MAZ transcription to local inflammation is warranted.

The differentially regulated network developed here elucidates differences between a low pathogenic and a moderately pathogenic infection, and is likely a suitable model of enhanced pathology in humans, as macaque models of influenza virus infection are considered to be one of the best surrogates of human infection [48]. Several chemokines and interleukins that are up-regulated in the CA04 infection are also up-regulated in the lungs of macaques infected with HPAI H5N1 virus [12]; however, promoter enrichment analysis of avian virus-infected lung tissue may be needed to provide greater clarity on the precise mechanisms active during a highly pathogenic infection. Ultimately, we intend to develop a mathematical model that can quickly identify the correlates of pathogenicity from microarray experiments to equate transcriptional regulation to infection severity in humans. The network presented here is the first step toward developing such a model.

## Conclusions

In summary, CA04-infected macaque lungs showed a prolonged immune response that continued beyond the duration of the local virus infection. The failure of the negative feedback mechanism that exists between MAZ, CDKN1A, and macrophage cell migration (induced by CCL2) could have caused this prolonged inflammation, thereby promoting enhanced cell cycle arrest and apoptosis. The interplay between MAZ induction, immune cell signaling, and inflammation must be finely tuned, and failure to maintain an appropriate, balanced response between these three factors could explain, at least in part, the increased pathogenicity of CA04 and other pH1N1 viruses. Further studies are needed to address the interchange between MAZ, the cell cycle, and the immune response, as well as the effects of this interplay on influenza virus-induced disease pathology. Overall, our strategy of linking functional annotations to the protein-protein interaction networks is suitable for identifying the key mechanisms driving the observed phenotypes.

## Methods

### Ethics statement

As previously reported in [11], all experiments were performed in accordance to the Guidelines for the Husbandry and Management of Laboratory Animals of the Research Center for Animal Life Science at Shiga University of Medical Science, Shiga, Japan and were approved by the Shiga University of Medical Science’s Animal Experiment Committee and Biosafety Committee.

### Tissue samples

Lung tissues used for microarray studies were obtained from thirteen female cynomolgus macaques infected with influenza viruses as previously described [11]. Briefly, six animals were inoculated with influenza A/California/04/2009 (H1N1; referred to as CA04), a 2009 pH1N1 virus isolate; six animals were inoculated with influenza A/Kawasaki/UTK-4/2009 (H1N1; referred to as KUTK4), a seasonal isolate; and one uninfected animal served as a negative control. On days 3 and 7 post-infection (p.i.), lung tissues were harvested from the middle and lower lung lobes of three animals in each infection group (N = 26 total samples were collected), and all but three samples were collected from visually apparent virus-induced gross lesions. Two lung tissue samples were obtained from middle and lower lobes of the uninfected animal at the start of the experiment. A more detailed description of tissue sample location and lesion severity is provided in Additional file 3.

### RNA extraction

Macaque lung tissues were placed in the RNA stabilization reagent RNAlater (Ambion, CA) and stored at -80°C. All tissues were thawed together and homogenized (2 minutes at 30 Hz) by using a TissueLyser (Qiagen, Hilden, Germany), following the manufacturer’s instructions. Total RNA was extracted from homogenized lung tissues with the RNeasy Mini Kit (Qiagen, Hilden, Germany), according to the manufacturer’s recommendation.

### Microarray, normalization, and statistical analysis

Extracted lung RNAs were sent to Takara Bio (Otsu, Shiga, Japan) for microarray analysis. Briefly, sample integrity and quantity were measured with the Agilent 2100 Bioanalyzer (Otsu, Shiga, Japan), which resulted in the exclusion of three samples due to poor RNA quality. In total, two mock-infected samples and at least five infected samples from each infection group at each time point were sent for subsequent microarray analysis. Cy3-labeled cRNA preparations were hybridized with rhesus macaque arrays (Agilent Microarray Design Identification Number 015421) for 17 h at 65°C. Feature Extraction Software version 7 (Agilent Technologies) was used for image analysis and data extraction, and Takara Bio provided whole array quality control metrics.

Per chip probe intensity normalization and differential expression (DE; DE also denotes “differentially expressed”, e.g., DE genes) analysis was performed by using GeneSpring GX version 11.0.2 (Agilent Technologies). Individual probe quality control was performed by using the GeneSpring default flag settings, requiring each probe to satisfy the flag conditions for at least 4 of the 25 samples. DE genes were identified between virus infection groups by use of one-way ANOVA complemented with a Tukey Honestly Significant Difference test, followed by a False Discovery Rate (FDR) correction (Benjamini-Hochberg). Criteria for DE were as follows: an absolute fold change > 2 and an FDR-adjusted *P*-value < 0.01. All microarray data have been deposited Gene Expression Omnibus (series number GSE39018) in accordance with Minimum Information About a Microarray Experiment (MIAME) guidelines.

### Ingenuity pathway analysis

Functional and pathway enrichment analyses were performed with Ingenuity Pathway Analysis (IPA) software (Ingenuity Systems, Redwood, CA, USA), after matching DE macaque genes with their human orthologs by using GenBank Accession identification numbers. For all functional and pathway enrichments, we required the Benjamini-Hochberg corrected *P*-value to be < 0.05. For cell-type specific functional enrichment analysis, significant function annotations were separated into their function and respective cell types (e.g., ‘recruitment of neutrophils’ was split into its function, ‘recruitment’, and the cell-type, ‘neutrophil’). Further, functions that were related were grouped together (e.g., ‘Cell Movement’, ‘Chemotaxis’, and ‘Trafficking’); thus, one cell-type may be significant more than once in each category. Additionally, leukocytes and mononuclear leukocytes were grouped together. Many of the cellular processes (e.g., *chemotaxis, trafficking* and *cell movement*) are highly related, we grouped them accordingly. Instances in which the same cell type is categorized into two cellular states (e.g., annotations related specifically to memory T cells or the more broad characterization of simply a T cell) were generalized to *T cells*. Both of these factors can make a single cell type appear enriched multiple times in each functional category. For each day, genes that were up-regulated or down-regulated when comparing CA04-infected lung tissue to KUTK4-infected lung tissue were analyzed separately.

### DAVID gene ontology analysis

The Genbank accession numbers of genes which were up or down-regulated when comparing CA04-infected lung tissue to KUTK4-infected tissue were analyzed in DAVID [15,49] using DAVID default settings. In addition to the enrichment scores for each annotation cluster produced by DAVID, we also determined the cluster size; i.e., the number of individual annotations which satisfied an FDR-adjusted P-value <0.01.

### Transcription factor promoter sequence enrichment analysis

The Genbank accession numbers of DE genes were analyzed in the GATHER [27] website to identify transcription factors whose promoter sites were highly represented within the data. GATHER employs the TRANSFAC 8.2 database, which contains data on transcription factors and their experimentally validated binding sites. The human genome R17 from the UCSC Genome Database was matched to high quality matrices for vertebrate regulator elements by applying the default score thresholds recommended by TRANSFAC. Transcription factor binding sites found 1200 bases upstream and 200 bases downstream of an annotated transcription start site were linked to their RefSeq IDs, which were then mapped to their Entrez Gene IDs based on the cross-references in the Entrez Gene database. GATHER scores the enrichment by using a *P*-value developed from the distribution of Bayes factors developed from randomly sampling 10,000 genes. Significant transcription factors were required to have an adjusted *P* < 0.05. Full details of the algorithm and justification for using the Bayes-based *P*-value is available in the original GATHER publication [27].

### Subnetwork construction

All subnetworks were constructed from the IPA PPI network using IPA’s internal algorithm. Briefly, the IPA algorithm identifies subnetworks by optimizing the interconnectivity and number of user genes (genes DE on each day) under the constraint of the selected network size. For the studies described here, we limited subnetworks to experimentally validated interactions identified in humans, and we performed iterative analysis of networks restricted to 35 or 70 total members. The degree of enrichment of DE genes in each subnetwork was indicated by the -log_10_ of the right-tailed Fisher’s Exact Test). Networks were constructed for each day separately.

### Network integration

The results from the promoter sequence enrichment analysis and pathway enrichment analysis were integrated by using protein-protein interaction data with the IPA interaction database. Stringent conditions were applied to identify binding interactions between the protein MAZ and two highly enriched, canonical pathways, namely “Role of CHK Proteins in Cell Cycle Checkpoint Control” and “Role of Cytokines in Mediating Communication between Immune Cells.” We required all added protein-protein interactions between these two networks to be in the Ingenuity Expert Findings and Ingenuity ExpertAssist Findings, and further required that the interaction had been verified in human lungs by using the setting within the IPA software. For completeness, we also included the viral RNA sensing pathway, as described in [40], to illustrate IRF7 activity on day 3 p.i.

### Statistical analysis

All tests for significant overlap between two gene lists were done in R using the one-sided Fisher’s exact test for enrichment. When determining the overlap between genes annotated with a select IPA term and genes containing a selecting binding sequence (as determined by GATHER), the gene symbols were first converted into unique gene identifiers using the DAVID gene ID converter.

## Competing interests

No competing interests to declare.

## Authors’ contributions

JES performed the enrichment, network and statistical analyses and drafted the manuscript. SF and AJE revised the manuscript and interpreted enrichment results. YM performed the RNA isolation and microarray development. SW and TW reviewed sample quality and participated in the project design. YM revised the manuscript and participated in the network development. HK and YK designed the project and revised the manuscript. All authors have read and approved the final manuscript.

## Supplementary Material

Additional file 1 **Summary of the number of probes DE and the number of DE probes which are chemokine ligands and receptors (CCL/R), interleukins (IL) or interferon stimulated genes for each day. Numbers in parenthesis show the number of up (left) and down regulated genes (right).** We also show the number of genes DE on both days (intersection). Click here for file

Additional file 2 All genes found differentially expressed on day 3 or 7 post infection. Click here for file

Additional file 3 Microarray data description file. File describes: from which lobe the RNA was isolated; which virus the animal was infected with; the day the sample was collected; the severity of the lesions from which the sample was isolated, and the amount of virus isolated from the region. Click here for file

Additional file 4 **Work Book Explanation: all genes lists were separated into up- or down-regulated when comparing CA04-infected tissue to KUTK4-infected.** DE gene's Genbank Accession IDs were uploaded into Ingenuity and the benjamini hochberg corrected P-value was used to quantify enrichment. Click here for file

Additional file 5 **Work Book Explanation: all genes lists were separated into up- or down-regulated when comparing CA04-infected tissue to KUTK4-infected.** DE gene's Genbank Accession IDs were uploaded into DAVID and the benjamini hochberg corrected P-value was used to quantify enrichment. Click here for file

Additional file 6 **Work Book Explanation: cell specific gene ontology enrichment. All genes lists were separated into up- or down-regulated when comparing CA04-infected tissue to KUTK4-infected.** DE gene's Genbank Accession ID was uploaded into Ingenuity and the benjamini hochberg corrected P-value was used to quantify enrichment. Here we report all categories with a FDR adjusted P-value < 0.05. Click here for file

Additional file 7 **Cell-specific CA04-induced functional enrichment on day 3 PI.** This is an enlarged illustration of Figure 3A which provides information on the specific function of each enriched IPA annotation. Click here for file

Additional file 8 **Cell-specific CA04-induced functional enrichment on day 7 PI.** This is an enlarged illustration of Figure 3B which provides information on the specific function of each enriched IPA annotation. Click here for file

Additional file 9 **The subnetwork of the human PPI which contains 70 proteins whose transcripts were significantly expressed in CA04-infected tissue.** This network was identified using IPA. Up-regulated genes are colored red while down-regulated genes are colored green. Click here for file

Additional file 10 **The "Role of Cytokines in Mediating Communication between Immune Cells" Pathway from the IPA database. Proteins colored red were identified as upregulated in CA04-infected tissue.** Interactions which promote protein production or cell proliferation are illustrates with arrows. Inhibitory interactions are illustrated with ┴. Click here for file

Additional file 11 **The "Role of CHK Proteins in Cell Cycle" Pathway from the IPA database.** Proteins colored red were identified as upregulated in CA04-infected tissue. Interactions which promote protein production or cell proliferation are illustrates with arrows. Inhibitory interactions are illustrated with ┴. Interactions which promote a particular phenotype (e.g., G2/S arrest) are illustrated with lines ending in a circle. Click here for file

Additional file 12 Work Book Explanation: enriched canonical biological pathways for genes differentially expressed between CA04 and KUTK4 infections. Click here for file

Additional file 13 **Work Book Explanation: official names, symbols and accession numbers of all proteins shown in Figure **5**.** Click here for file
