# Supplementary material for: Integrated network analysis reveals a novel role for the cell cycle in 2009 pandemic influenza virus-induced inflammation in macaque lungs
Source: BMC Syst Biol. 2012 Aug 31;6:117. doi: 10.1186/1752-0509-6-117 (PMC3481363; doi:10.1186/1752-0509-6-117)
Supplement: Additional file 7 — Cell-specific CA04-induced functional enrichment on day 3 PI. This is an enlarged illustration of Figure 3A which provides information on the specific function of each enriched IPA annotation. [file 1752-0509-6-117-S7.pdf]

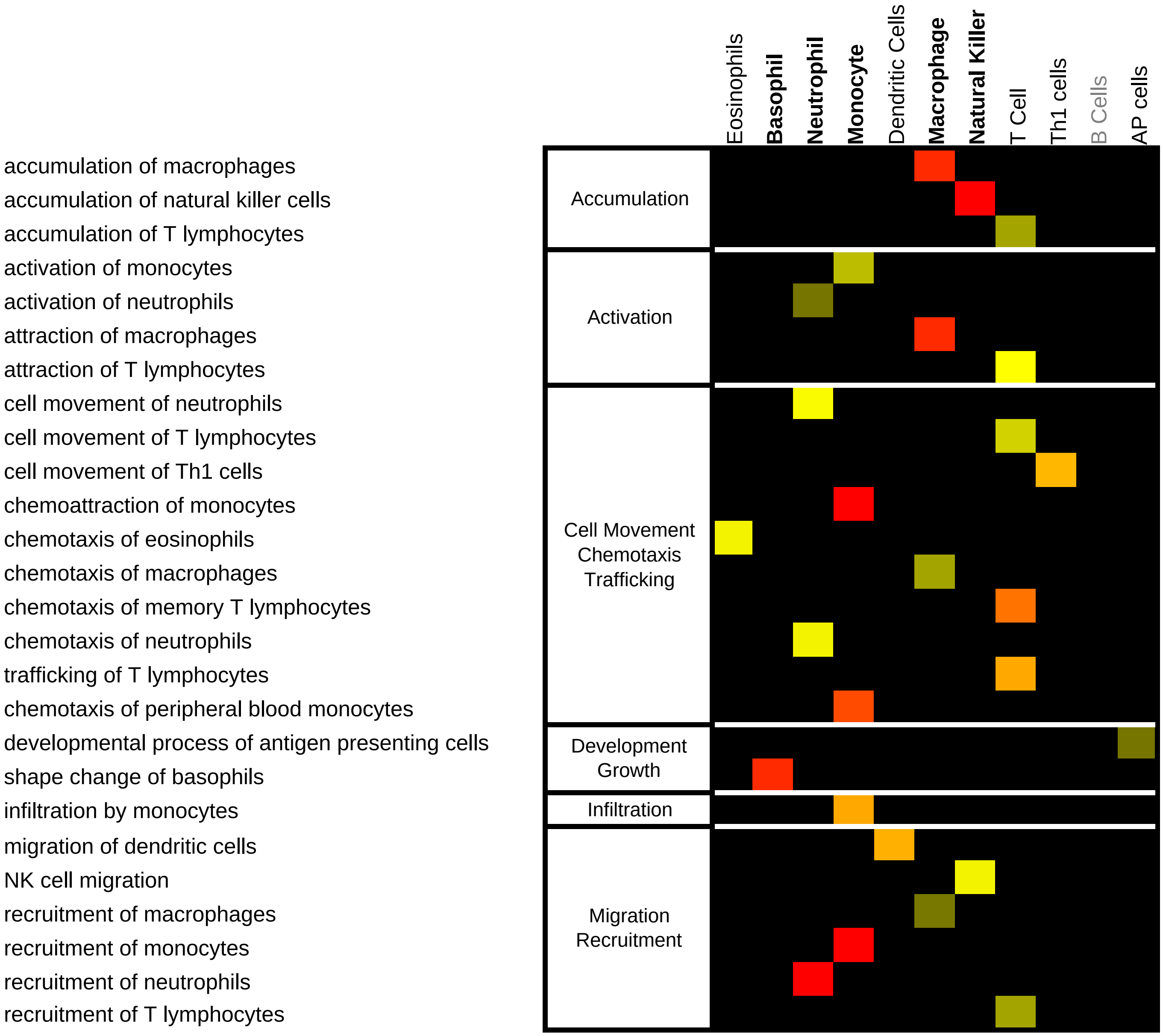
Eosinophils
Basophil
Neutrophil
Monocyte
Dendritic Cells
Macrophage
Natural Killer
T Cell
Th1 cells
B Cells
AP cells
accumulation of macrophages
accumulation of natural killer cells
accumulation of T lymphocytes
activation of monocytes
activation of neutrophils
attraction of macrophages
attraction of T lymphocytes
cell movement of neutrophils
cell movement of T lymphocytes
cell movement of Th1 cells
chemoattraction of monocytes
chemotaxis of eosinophils
chemotaxis of macrophages
chemotaxis of memory T lymphocytes
chemotaxis of neutrophils
trafficking of T lymphocytes
chemotaxis of peripheral blood monocytes
developmental process of antigen presenting cells
shape change of basophils
infiltration by monocytes
migration of dendritic cells
NK cell migration
recruitment of macrophages
recruitment of monocytes
recruitment of neutrophils
recruitment of T lymphocytes
Accumulation
Activation
Cell Movement
Chemotaxis
Trafficking
Development
Growth
Infiltration
Migration
Recruitment
