# Supplementary material for: Integrated network analysis reveals a novel role for the cell cycle in 2009 pandemic influenza virus-induced inflammation in macaque lungs
Source: BMC Syst Biol. 2012 Aug 31;6:117. doi: 10.1186/1752-0509-6-117 (PMC3481363; doi:10.1186/1752-0509-6-117)
Supplement: Additional file 8 — Cell-specific CA04-induced functional enrichment on day 7 PI. This is an enlarged illustration of Figure 3B which provides information on the specific function of each enriched IPA annotation. [file 1752-0509-6-117-S8.pdf]

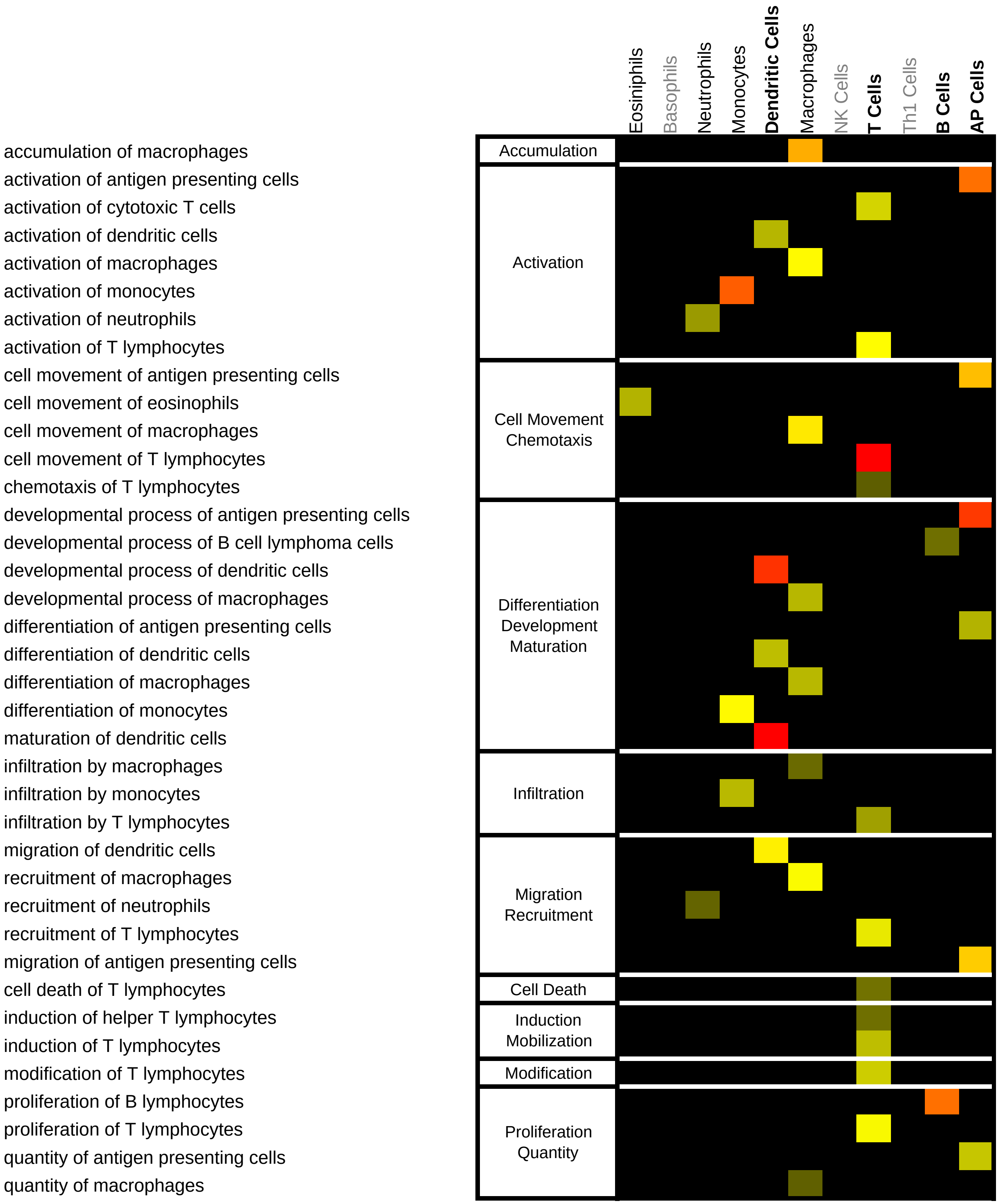
Eosiniphils
Basophils
Neutrophils
Monocytes
Dendritic Cells
Macrophages
NK Cells
T Cells
Th1 Cells
B Cells
AP Cells
Accumulation
Activation
Cell Movement Chemotaxis
Differentiation Development Maturation
Infiltration
Migration Recruitment
Cell Death
Induction Mobilization
Modification
Proliferation Quantity
accumulation of macrophages
activation of antigen presenting cells
activation of cytotoxic T cells
activation of dendritic cells
activation of macrophages
activation of monocytes
activation of neutrophils
activation of T lymphocytes
cell movement of antigen presenting cells
cell movement of eosinophils
cell movement of macrophages
cell movement of T lymphocytes
chemotaxis of T lymphocytes
developmental process of antigen presenting cells
developmental process of B cell lymphoma cells
developmental process of dendritic cells
developmental process of macrophages
differentiation of antigen presenting cells
differentiation of dendritic cells
differentiation of macrophages
differentiation of monocytes
maturation of dendritic cells
infiltration by macrophages
infiltration by monocytes
infiltration by T lymphocytes
migration of dendritic cells
recruitment of macrophages
recruitment of neutrophils
recruitment of T lymphocytes
migration of antigen presenting cells
cell death of T lymphocytes
induction of helper T lymphocytes
induction of T lymphocytes
modification of T lymphocytes
proliferation of B lymphocytes
proliferation of T lymphocytes
quantity of antigen presenting cells
quantity of macrophages
