# Supplementary figures and images for: Integrated network analysis reveals a novel role for the cell cycle in 2009 pandemic influenza virus-induced inflammation in macaque lungs
Source: BMC Syst Biol. 2012 Aug 31;6:117. doi: 10.1186/1752-0509-6-117 (PMC3481363; doi:10.1186/1752-0509-6-117)

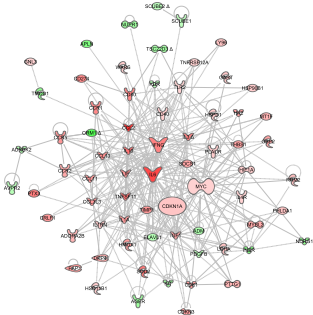

Supplement: Additional file 9 — The subnetwork of the human PPI which contains 70 proteins whose transcripts were significantly expressed in CA04-infected tissue. This network was identified using IPA. Up-regulated genes are colored red while down-regulated genes are colored green. [file 1752-0509-6-117-S9.pdf]

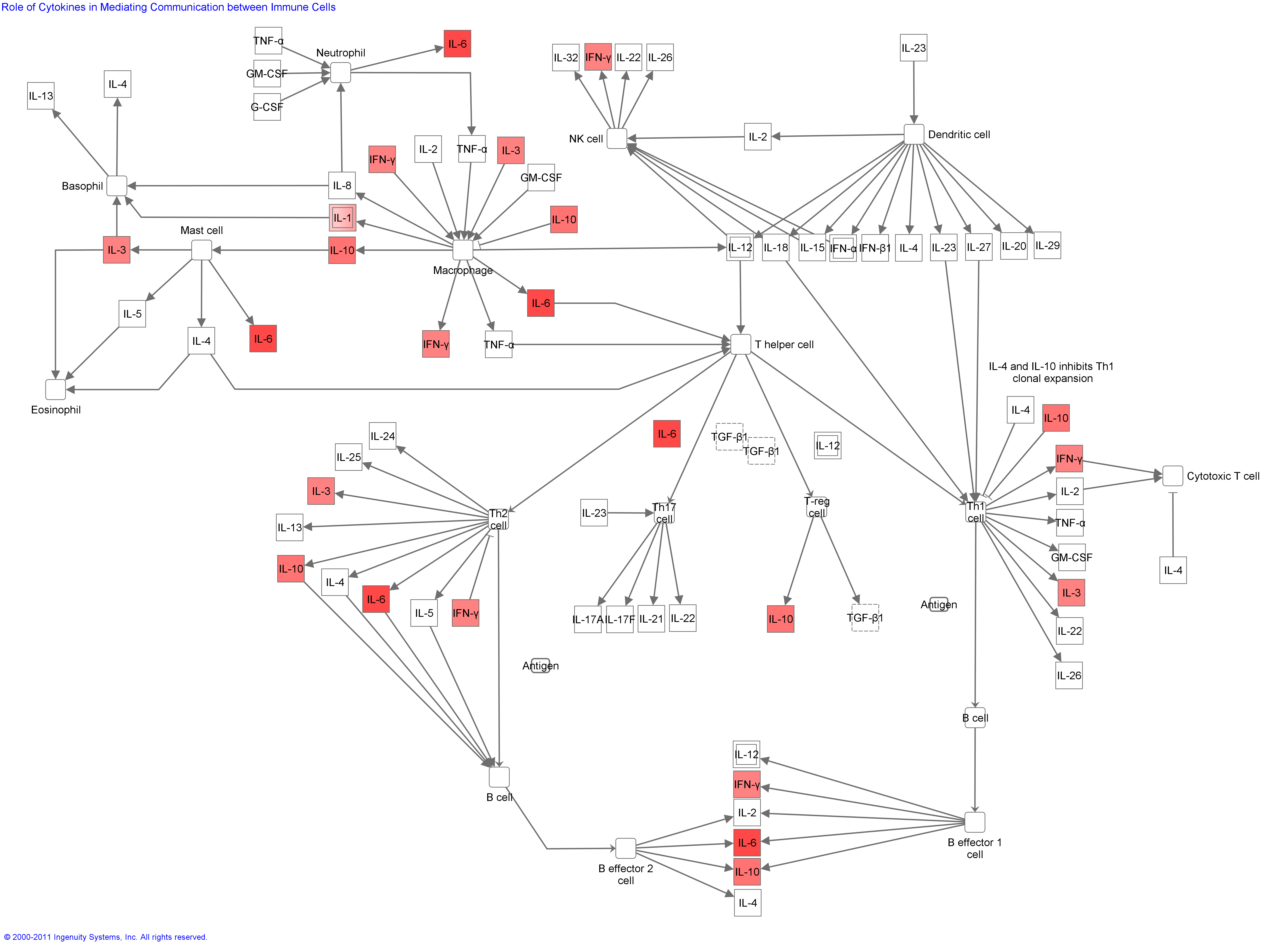

Supplement: Additional file 10 — The "Role of Cytokines in Mediating Communication between Immune Cells" Pathway from the IPA database. Proteins colored red were identified as upregulated in CA04-infected tissue. Interactions which promote protein production or cell proliferation are illustrates with arrows. Inhibitory interactions are illustrated with ┴. [file 1752-0509-6-117-S10.png]

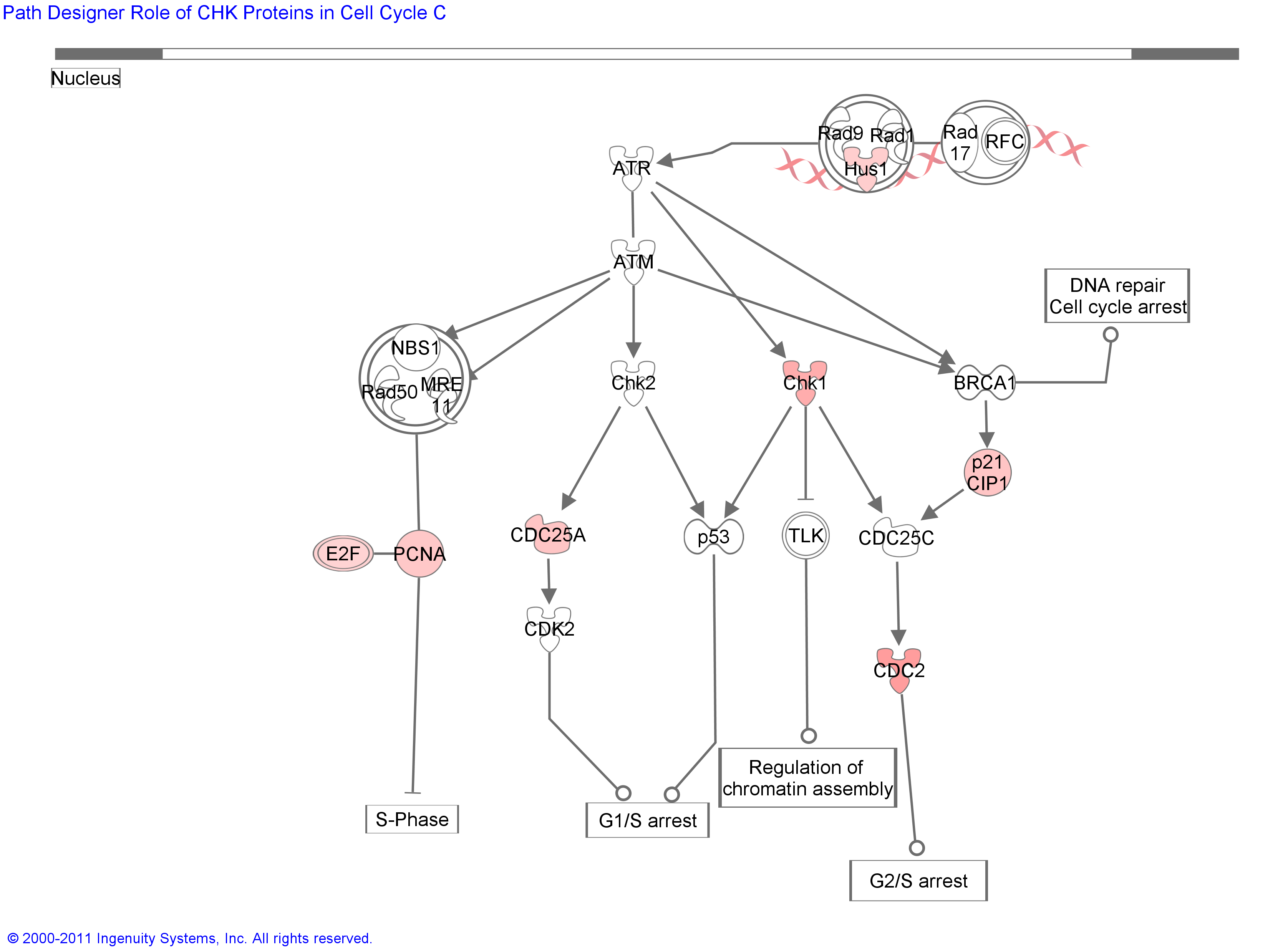

Supplement: Additional file 11 — The "Role of CHK Proteins in Cell Cycle" Pathway from the IPA database. Proteins colored red were identified as upregulated in CA04-infected tissue. Interactions which promote protein production or cell proliferation are illustrates with arrows. Inhibitory interactions are illustrated with ┴. Interactions which promote a particular phenotype (e.g., G2/S arrest) are illustrated with lines ending in a circle. [file 1752-0509-6-117-S11.png]
